# Ethyl 2-[4-(benzyloxy)anilino]-4-oxo-4,5-dihydro­furan-3-carboxyl­ate

**DOI:** 10.1107/S1600536808035988

**Published:** 2008-11-13

**Authors:** S. Nirmala, R. Murugan, E. Theboral Sugi Kamala, L. Sudha, S. Sriman Narayanan

**Affiliations:** a Department of Physics, Easwari Engineering College, Ramapuram, Chennai 600 089, India; b Department of Analytical Chemistry, University of Madras, Guindy Campus, Chennai 600 025, India; c Department of Physics, SRM University, Ramapuram Campus, Chennai 600 089, India

## Abstract

In the title compound, C_20_H_19_NO_5_, the dihydro­furan ring is almost planar [maximum deviation of 0.021 (2)°] and makes dihedral angles of 28.1 (7) and 54.5 (5)° with the benzyl and phenyl­amino rings, respectively. The mol­ecular packing is stabilized by intra­molecular N—H⋯O hydrogen bonds and inter­molecular C—H⋯O inter­actions.

## Related literature

For background on the development of effective and tolerable therapeutic options for cervical cancer, see: Huang *et al.* (2007 ▶); Lu *et al.* (2008 ▶). For the analysis of apoptosis induced by dihydro­furan carboxyl­ate compounds, see: Chen *et al.* (2006 ▶); Lin *et al.* (2006 ▶); Zhang & Wei (2007 ▶). For bond-length data, see: Allen *et al.* (1987 ▶). For a related structure, see: Erdsack *et al.* (2007 ▶).
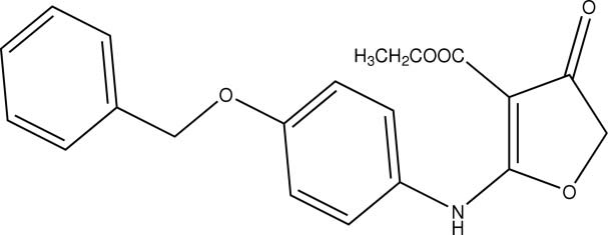


## Experimental

### 

#### Crystal data

C_20_H_19_NO_5_
*M* *_r_* = 353.36
Triclinic, 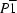

*a* = 9.1315 (3) Å
*b* = 10.4040 (3) Å
*c* = 11.1162 (4) Å
α = 84.848 (2)°
β = 66.436 (2)°
γ = 64.121 (2)°
*V* = 866.34 (5) Å^3^
*Z* = 2
Mo *K*α radiation
μ = 0.10 mm^−1^
*T* = 293 (2) K
0.25 × 0.20 × 0.20 mm

#### Data collection

Bruker Kappa APEXII diffractometer
Absorption correction: multi-scan (Blessing, 1995 ▶) *T*_min_ = 0.976, *T*_max_ = 0.981
22596 measured reflections
5349 independent reflections
3665 reflections with *I* > 2σ(*I*)
*R*_int_ = 0.025

#### Refinement

*R*[*F* ^2^ > 2σ(*F* ^2^)] = 0.051
*wR*(*F* ^2^) = 0.163
*S* = 1.03
5349 reflections
235 parameters
H-atom parameters constrained
Δρ_max_ = 0.29 e Å^−3^
Δρ_min_ = −0.20 e Å^−3^

### 

Data collection: *APEX2* (Bruker, 2004 ▶); cell refinement: *APEX2* and *SAINT* (Bruker, 2004 ▶); data reduction: *SAINT* and *XPREP* (Bruker, 2004 ▶); program(s) used to solve structure: *SHELXS97* (Sheldrick, 2008 ▶); program(s) used to refine structure: *SHELXL97* (Sheldrick, 2008 ▶); molecular graphics: *ORTEP-3* (Farrugia, 1997 ▶); software used to prepare material for publication: *PLATON* (Spek, 2003 ▶).

## Supplementary Material

Crystal structure: contains datablocks I, global. DOI: 10.1107/S1600536808035988/bq2103sup1.cif

Structure factors: contains datablocks I. DOI: 10.1107/S1600536808035988/bq2103Isup2.hkl

Additional supplementary materials:  crystallographic information; 3D view; checkCIF report

## Figures and Tables

**Table 1:** Hydrogen-bond geometry (Å, °)

*D*—H⋯*A*	*D*—H	H⋯*A*	*D*⋯*A*	*D*—H⋯*A*
N1—H1⋯O4	0.86	2.12	2.7485 (15)	129
C6—H6⋯O3^i^	0.93	2.51	3.3951 (18)	160
C17—H17⋯O4^ii^	0.93	2.58	3.465 (2)	160

Symmetry codes: (i) 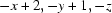; (ii) 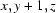.

**Fig. 1.**
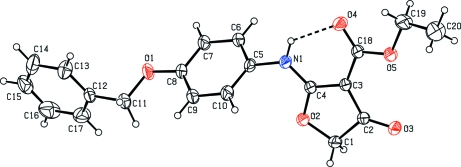
The molecular structure of the title compound with 30% probability displacement ellipsoids. Dashed line indicates hydrogen bond.

**Fig. 2.**
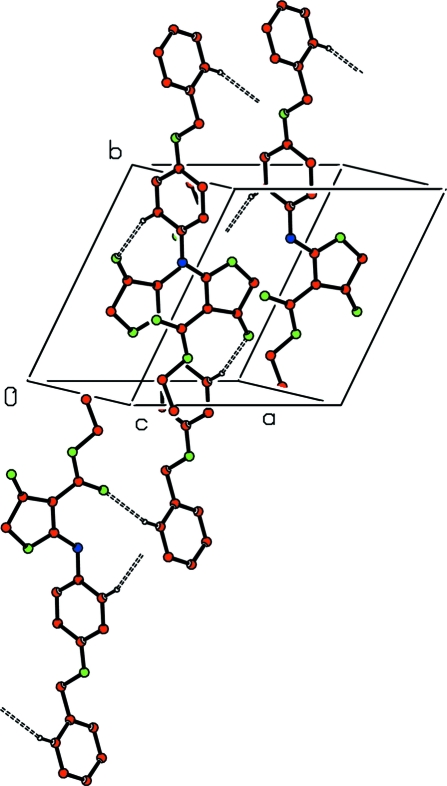
The packing of the molecules viewed down the c axis. Dashed lines indicate hydrogen bonds. H atoms not involed in hydrogen bonds have been omitted.

## supplementary crystallographic information

### Comment

Human cervical cancer is potentially lethal, and therefore the development of effective and tolerable therapeutic options is vital (Huang *et al.*, 2007; Lu *et al.*, 2008). Dihydrofuran carboxylate compounds induced morphological changes and cytotoxicity in a dose - dependent manner. Dihydrofuran carboxylate compounds induced apoptosis which was analyzed by flow cytometric methods and confirmed by DAPI staining and DNA fragmentation analyzed by DNA gel electrophoresis (Chen *et al.*, 2006; Lin *et al.*, 2006; Zhang & Wei, 2007). In view of this medicinal importance, an X-ray study of the title compound, (I), was carried out.

An *ORTEP *(Farrugia,1997) plot of the molecule is shown in Fig. 1. The bond lengths in (I) show normal values (Allen *et al.*, 1987) and are comparable to the related structure (Erdsack *et al.*, 2007). The dihydrofuran ring (O2/C1—C4) is planar with a maximum deviation of -0.021 (2)° for C3 from the least square plane defined by all non hydrogen atoms in the molecule. The dihydrofuran ring makes dihedral angles of 28.1 (7)° and 54.5 (5)°, respectively, with the benzyl ring (C12—C17) and phenylamino ring (C5—C10), whereas the benzyl and phenylamino rings are oriented at an angle of 78.6 (6)° with respect to each other.

The crystal structure is stabilized by intramolecular N—H···O interactions. In addition to the van der Waals interactions, the molecular packing in the crystal is also stabilized by intermolecular C—H···O interactions (Table 1, Fig. 2).

### Experimental

1.0 mol of 4-(benzyloxy) aniline (1.0 g) and 1.0 mol of ethyl 2-chloro-4- oxo-4,5-dihydrofuran-3-carboxylate (0.9 g) was allowed to stir in 10 ml of dichloromethane which contains 0.5 ml of triethylamine at room temperature for about 8 hrs. The completion of the reaction was monitored by TLC. After the completion of reaction the crude solid was filtered and then recrystallized in ethanol.

### Refinement

H atoms were positioned geometrically and were treated as riding on their parent C atoms, with aromatic C—H distances of 0.93 Å, methyl C—H distances of 0.96 Å and methylene C—H distances of 0.97 Å, and with *U*_iso_(H) = 1.5*U*_eq_(C) for methyl H and 1.2*U*_eq_(C) for other H atoms.

### Crystal data

**Table:**

C_20_H_19_NO_5_	*Z* = 2
*M**_r_* = 353.36	*F*_000_ = 372
Triclinic, *P*1	*D*_x_ = 1.355 Mg m^−^^3^
Hall symbol: -P 1	Mo *K*α radiation λ = 0.71073 Å
*a* = 9.1315 (3) Å	Cell parameters from 6361 reflections
*b* = 10.4040 (3) Å	θ = 2.6–30.7º
*c* = 11.1162 (4) Å	µ = 0.10 mm^−^^1^
α = 84.848 (2)º	*T* = 293 (2) K
β = 66.436 (2)º	Prism, yellow
γ = 64.121 (2)º	0.25 × 0.20 × 0.20 mm
*V* = 866.34 (5) Å^3^	

### Data collection

**Table:**

Bruker Kappa APEXII diffractometer	5349 independent reflections
Radiation source: fine-focus sealed tube	3665 reflections with *I* > 2σ(*I*)
Monochromator: graphite	*R*_int_ = 0.025
*T* = 293(2) K	θ_max_ = 30.7º
Bruker axs (kappa apex2) scans	θ_min_ = 2.0º
Absorption correction: multi-scan(Blessing, 1995)	*h* = −13→13
*T*_min_ = 0.976, *T*_max_ = 0.981	*k* = −14→14
22596 measured reflections	*l* = −15→15

### Refinement

**Table:**

Refinement on *F*^2^	Secondary atom site location: difference Fourier map
Least-squares matrix: full	Hydrogen site location: inferred from neighbouring sites
*R*[*F*^2^ > 2σ(*F*^2^)] = 0.051	H-atom parameters constrained
*wR*(*F*^2^) = 0.163	*w* = 1/[σ^2^(*F*_o_^2^) + (0.0812*P*)^2^ + 0.1488*P*] where *P* = (*F*_o_^2^ + 2*F*_c_^2^)/3
*S* = 1.03	(Δ/σ)_max_ < 0.001
5349 reflections	Δρ_max_ = 0.29 e Å^−^^3^
235 parameters	Δρ_min_ = −0.20 e Å^−^^3^
Primary atom site location: structure-invariant direct methods	Extinction correction: none

### Special details

**Table:**

Geometry. All e.s.d.'s (except the e.s.d. in the dihedral angle between two l.s. planes) are estimated using the full covariance matrix. The cell e.s.d.'s are taken into account individually in the estimation of e.s.d.'s in distances, angles and torsion angles; correlations between e.s.d.'s in cell parameters are only used when they are defined by crystal symmetry. An approximate (isotropic) treatment of cell e.s.d.'s is used for estimating e.s.d.'s involving l.s. planes.
Refinement. Refinement of *F*^2^ against ALL reflections. The weighted *R*-factor *wR* and goodness of fit *S* are based on *F*^2^, conventional *R*-factors *R* are based on *F*, with *F* set to zero for negative *F*^2^. The threshold expression of *F*^2^ > σ(*F*^2^) is used only for calculating *R*-factors(gt) *etc*. and is not relevant to the choice of reflections for refinement. *R*-factors based on *F*^2^ are statistically about twice as large as those based on *F*, and *R*- factors based on ALL data will be even larger.

### Fractional atomic coordinates and isotropic or equivalent isotropic displacement parameters (Å^2^)

**Table:**

	*x*	*y*	*z*	*U*_iso_*/*U*_eq_	
C1	1.26357 (19)	0.57501 (15)	0.0775 (2)	0.0570 (4)	
H1A	1.3252	0.5714	0.1323	0.068*	
H1B	1.3224	0.6003	−0.0086	0.068*	
C2	1.26228 (18)	0.43137 (14)	0.06491 (15)	0.0437 (3)	
C3	1.07887 (17)	0.46034 (12)	0.12356 (14)	0.0386 (3)	
C4	0.98080 (17)	0.60818 (13)	0.15886 (14)	0.0396 (3)	
C5	0.71086 (17)	0.83503 (13)	0.24252 (14)	0.0421 (3)	
C6	0.58376 (19)	0.91391 (14)	0.19371 (16)	0.0496 (3)	
H6	0.5628	0.8684	0.1383	0.060*	
C7	0.4878 (2)	1.06059 (15)	0.22738 (17)	0.0517 (4)	
H7	0.4011	1.1138	0.1956	0.062*	
C8	0.52074 (18)	1.12851 (14)	0.30861 (15)	0.0447 (3)	
C9	0.6476 (2)	1.04905 (15)	0.35756 (16)	0.0515 (4)	
H9	0.6699	1.0941	0.4122	0.062*	
C10	0.7412 (2)	0.90186 (15)	0.32472 (16)	0.0513 (4)	
H10	0.8253	0.8479	0.3587	0.062*	
C11	0.4551 (2)	1.34824 (16)	0.41338 (19)	0.0572 (4)	
H11A	0.5762	1.3371	0.3698	0.069*	
H11B	0.4396	1.3104	0.4983	0.069*	
C12	0.3260 (2)	1.50396 (14)	0.43163 (15)	0.0464 (3)	
C13	0.1520 (2)	1.54983 (19)	0.51716 (18)	0.0639 (4)	
H13	0.1136	1.4836	0.5635	0.077*	
C14	0.0329 (3)	1.6920 (2)	0.5359 (2)	0.0798 (6)	
H14	−0.0853	1.7216	0.5940	0.096*	
C15	0.0884 (3)	1.78924 (19)	0.4692 (3)	0.0816 (7)	
H15	0.0083	1.8858	0.4818	0.098*	
C16	0.2604 (4)	1.7455 (2)	0.3842 (3)	0.0888 (7)	
H16	0.2983	1.8124	0.3390	0.107*	
C17	0.3794 (3)	1.6028 (2)	0.3642 (2)	0.0678 (5)	
H17	0.4968	1.5734	0.3045	0.081*	
C18	0.99189 (18)	0.36906 (13)	0.14162 (14)	0.0416 (3)	
C19	1.0226 (3)	0.13250 (17)	0.1330 (3)	0.0729 (6)	
H19A	0.9252	0.1571	0.2193	0.088*	
H19B	0.9755	0.1409	0.0669	0.088*	
C20	1.1542 (3)	−0.01258 (19)	0.1230 (3)	0.0969 (8)	
H20A	1.1010	−0.0772	0.1362	0.145*	
H20B	1.1996	−0.0205	0.1892	0.145*	
H20C	1.2498	−0.0367	0.0372	0.145*	
N1	0.80732 (15)	0.68332 (11)	0.20754 (13)	0.0465 (3)	
H1	0.7456	0.6366	0.2196	0.056*	
O1	0.42051 (15)	1.27356 (10)	0.33465 (12)	0.0582 (3)	
O2	1.07889 (13)	0.67841 (9)	0.13778 (12)	0.0516 (3)	
O3	1.39676 (13)	0.32190 (11)	0.01100 (13)	0.0599 (3)	
O4	0.83217 (13)	0.41370 (11)	0.18030 (13)	0.0568 (3)	
O5	1.10361 (13)	0.22984 (10)	0.11310 (12)	0.0522 (3)	

### Atomic displacement parameters (Å^2^)

**Table:**

	*U*^11^	*U*^22^	*U*^33^	*U*^12^	*U*^13^	*U*^23^
C1	0.0359 (7)	0.0384 (7)	0.0945 (12)	−0.0144 (6)	−0.0230 (7)	−0.0096 (7)
C2	0.0369 (6)	0.0328 (6)	0.0618 (8)	−0.0125 (5)	−0.0221 (6)	−0.0017 (5)
C3	0.0350 (6)	0.0269 (5)	0.0514 (7)	−0.0118 (4)	−0.0164 (5)	0.0010 (5)
C4	0.0365 (6)	0.0293 (5)	0.0512 (7)	−0.0138 (5)	−0.0161 (5)	0.0010 (5)
C5	0.0329 (6)	0.0283 (5)	0.0550 (8)	−0.0099 (5)	−0.0111 (5)	−0.0017 (5)
C6	0.0440 (7)	0.0350 (6)	0.0669 (9)	−0.0108 (6)	−0.0241 (7)	−0.0080 (6)
C7	0.0477 (8)	0.0345 (6)	0.0683 (10)	−0.0062 (6)	−0.0297 (7)	−0.0062 (6)
C8	0.0396 (7)	0.0313 (6)	0.0531 (8)	−0.0074 (5)	−0.0159 (6)	−0.0064 (5)
C9	0.0499 (8)	0.0383 (7)	0.0627 (9)	−0.0108 (6)	−0.0261 (7)	−0.0081 (6)
C10	0.0473 (8)	0.0365 (7)	0.0638 (9)	−0.0073 (6)	−0.0275 (7)	−0.0013 (6)
C11	0.0551 (9)	0.0374 (7)	0.0748 (11)	−0.0102 (6)	−0.0299 (8)	−0.0108 (7)
C12	0.0490 (8)	0.0342 (6)	0.0518 (8)	−0.0126 (6)	−0.0204 (6)	−0.0068 (5)
C13	0.0594 (10)	0.0494 (9)	0.0610 (10)	−0.0147 (8)	−0.0135 (8)	0.0040 (7)
C14	0.0601 (11)	0.0616 (11)	0.0776 (13)	0.0030 (9)	−0.0178 (10)	−0.0184 (10)
C15	0.0951 (16)	0.0345 (8)	0.1246 (18)	−0.0077 (9)	−0.0732 (15)	−0.0061 (10)
C16	0.1034 (18)	0.0548 (11)	0.144 (2)	−0.0476 (12)	−0.0750 (17)	0.0374 (12)
C17	0.0592 (10)	0.0604 (10)	0.0887 (13)	−0.0320 (9)	−0.0288 (10)	0.0112 (9)
C18	0.0398 (7)	0.0289 (5)	0.0547 (8)	−0.0148 (5)	−0.0179 (6)	0.0041 (5)
C19	0.0681 (11)	0.0381 (8)	0.1251 (17)	−0.0328 (8)	−0.0411 (11)	0.0130 (9)
C20	0.0866 (15)	0.0382 (9)	0.163 (3)	−0.0326 (10)	−0.0444 (16)	0.0207 (12)
N1	0.0357 (6)	0.0280 (5)	0.0686 (8)	−0.0124 (4)	−0.0146 (5)	−0.0015 (5)
O1	0.0597 (7)	0.0308 (5)	0.0779 (8)	−0.0033 (4)	−0.0362 (6)	−0.0140 (5)
O2	0.0383 (5)	0.0299 (4)	0.0823 (8)	−0.0144 (4)	−0.0185 (5)	−0.0058 (4)
O3	0.0359 (5)	0.0388 (5)	0.0931 (9)	−0.0083 (4)	−0.0198 (5)	−0.0128 (5)
O4	0.0388 (5)	0.0388 (5)	0.0878 (8)	−0.0184 (4)	−0.0185 (5)	0.0037 (5)
O5	0.0450 (5)	0.0266 (4)	0.0821 (8)	−0.0160 (4)	−0.0215 (5)	0.0018 (4)

### Geometric parameters (Å, °)

**Table:**

C1—O2	1.4466 (17)	C11—H11A	0.9700
C1—C2	1.5190 (19)	C11—H11B	0.9700
C1—H1A	0.9700	C12—C17	1.365 (2)
C1—H1B	0.9700	C12—C13	1.367 (2)
C2—O3	1.2156 (16)	C13—C14	1.373 (2)
C2—C3	1.4291 (19)	C13—H13	0.9300
C3—C4	1.3953 (16)	C14—C15	1.357 (3)
C3—C18	1.4386 (17)	C14—H14	0.9300
C4—N1	1.3126 (17)	C15—C16	1.354 (4)
C4—O2	1.3281 (15)	C15—H15	0.9300
C5—C10	1.371 (2)	C16—C17	1.376 (3)
C5—C6	1.381 (2)	C16—H16	0.9300
C5—N1	1.4287 (15)	C17—H17	0.9300
C6—C7	1.3820 (18)	C18—O4	1.2128 (17)
C6—H6	0.9300	C18—O5	1.3316 (15)
C7—C8	1.387 (2)	C19—C20	1.441 (3)
C7—H7	0.9300	C19—O5	1.4506 (17)
C8—O1	1.3637 (15)	C19—H19A	0.9700
C8—C9	1.381 (2)	C19—H19B	0.9700
C9—C10	1.3847 (19)	C20—H20A	0.9600
C9—H9	0.9300	C20—H20B	0.9600
C10—H10	0.9300	C20—H20C	0.9600
C11—O1	1.4238 (18)	N1—H1	0.8600
C11—C12	1.5022 (19)		
			
O2—C1—C2	105.83 (11)	C17—C12—C13	118.54 (15)
O2—C1—H1A	110.6	C17—C12—C11	121.00 (15)
C2—C1—H1A	110.6	C13—C12—C11	120.46 (15)
O2—C1—H1B	110.6	C12—C13—C14	121.09 (18)
C2—C1—H1B	110.6	C12—C13—H13	119.5
H1A—C1—H1B	108.7	C14—C13—H13	119.5
O3—C2—C3	131.81 (12)	C15—C14—C13	119.7 (2)
O3—C2—C1	122.98 (13)	C15—C14—H14	120.1
C3—C2—C1	105.18 (11)	C13—C14—H14	120.1
C4—C3—C2	106.96 (11)	C16—C15—C14	119.92 (17)
C4—C3—C18	121.03 (12)	C16—C15—H15	120.0
C2—C3—C18	131.90 (11)	C14—C15—H15	120.0
N1—C4—O2	117.79 (11)	C15—C16—C17	120.4 (2)
N1—C4—C3	127.88 (12)	C15—C16—H16	119.8
O2—C4—C3	114.33 (11)	C17—C16—H16	119.8
C10—C5—C6	120.12 (12)	C12—C17—C16	120.32 (19)
C10—C5—N1	120.95 (13)	C12—C17—H17	119.8
C6—C5—N1	118.92 (13)	C16—C17—H17	119.8
C5—C6—C7	119.82 (13)	O4—C18—O5	122.88 (12)
C5—C6—H6	120.1	O4—C18—C3	123.59 (12)
C7—C6—H6	120.1	O5—C18—C3	113.52 (11)
C6—C7—C8	120.03 (14)	C20—C19—O5	109.29 (15)
C6—C7—H7	120.0	C20—C19—H19A	109.8
C8—C7—H7	120.0	O5—C19—H19A	109.8
O1—C8—C9	124.60 (13)	C20—C19—H19B	109.8
O1—C8—C7	115.48 (13)	O5—C19—H19B	109.8
C9—C8—C7	119.92 (12)	H19A—C19—H19B	108.3
C8—C9—C10	119.57 (14)	C19—C20—H20A	109.5
C8—C9—H9	120.2	C19—C20—H20B	109.5
C10—C9—H9	120.2	H20A—C20—H20B	109.5
C5—C10—C9	120.52 (14)	C19—C20—H20C	109.5
C5—C10—H10	119.7	H20A—C20—H20C	109.5
C9—C10—H10	119.7	H20B—C20—H20C	109.5
O1—C11—C12	107.46 (12)	C4—N1—C5	126.48 (11)
O1—C11—H11A	110.2	C4—N1—H1	116.8
C12—C11—H11A	110.2	C5—N1—H1	116.8
O1—C11—H11B	110.2	C8—O1—C11	117.26 (12)
C12—C11—H11B	110.2	C4—O2—C1	107.56 (10)
H11A—C11—H11B	108.5	C18—O5—C19	115.87 (12)
			
O2—C1—C2—O3	175.75 (15)	C12—C13—C14—C15	−0.4 (3)
O2—C1—C2—C3	−2.44 (18)	C13—C14—C15—C16	0.3 (3)
O3—C2—C3—C4	−174.22 (17)	C14—C15—C16—C17	0.4 (4)
C1—C2—C3—C4	3.74 (17)	C13—C12—C17—C16	1.0 (3)
O3—C2—C3—C18	1.9 (3)	C11—C12—C17—C16	−178.56 (18)
C1—C2—C3—C18	179.84 (16)	C15—C16—C17—C12	−1.1 (3)
C2—C3—C4—N1	174.99 (15)	C4—C3—C18—O4	4.4 (2)
C18—C3—C4—N1	−1.6 (2)	C2—C3—C18—O4	−171.19 (16)
C2—C3—C4—O2	−4.03 (17)	C4—C3—C18—O5	−174.86 (13)
C18—C3—C4—O2	179.36 (13)	C2—C3—C18—O5	9.5 (2)
C10—C5—C6—C7	0.3 (2)	O2—C4—N1—C5	−0.9 (2)
N1—C5—C6—C7	179.38 (14)	C3—C4—N1—C5	−179.88 (14)
C5—C6—C7—C8	0.8 (2)	C10—C5—N1—C4	−52.3 (2)
C6—C7—C8—O1	179.24 (15)	C6—C5—N1—C4	128.62 (17)
C6—C7—C8—C9	−1.1 (2)	C9—C8—O1—C11	2.8 (2)
O1—C8—C9—C10	179.81 (15)	C7—C8—O1—C11	−177.55 (15)
C7—C8—C9—C10	0.1 (3)	C12—C11—O1—C8	−178.33 (13)
C6—C5—C10—C9	−1.3 (2)	N1—C4—O2—C1	−176.74 (14)
N1—C5—C10—C9	179.71 (14)	C3—C4—O2—C1	2.39 (18)
C8—C9—C10—C5	1.0 (3)	C2—C1—O2—C4	0.15 (18)
O1—C11—C12—C17	−104.90 (19)	O4—C18—O5—C19	−0.9 (2)
O1—C11—C12—C13	75.5 (2)	C3—C18—O5—C19	178.40 (16)
C17—C12—C13—C14	−0.3 (3)	C20—C19—O5—C18	−168.96 (18)
C11—C12—C13—C14	179.31 (17)		

### Hydrogen-bond geometry (Å, °)

**Table:**

*D*—H···*A*	*D*—H	H···*A*	*D*···*A*	*D*—H···*A*
N1—H1···O4	0.86	2.12	2.7485 (15)	129
C6—H6···O3^i^	0.93	2.51	3.3951 (18)	160
C17—H17···O4^ii^	0.93	2.58	3.465 (2)	160

Symmetry codes: (i) −*x*+2, −*y*+1, −*z*; (ii) *x*, *y*+1, *z*.
